# miR-149 represses metastasis of hepatocellular carcinoma by targeting actin-regulatory proteins PPM1F

**DOI:** 10.18632/oncotarget.5676

**Published:** 2015-10-14

**Authors:** Gang Luo, Ya-Ling Chao, Bo Tang, Bo-Sheng Li, Yu-Feng Xiao, Rui Xie, Shu-Ming Wang, Yu-Yun Wu, Hui Dong, Xiang-De Liu, Shi-Ming Yang

**Affiliations:** ^1^ Department of Gastroenterology, Xinqiao Hospital, Third Military Medical University, Chongqing 400037, P.R. China; ^2^ Institute of Hepatobiliary Surgery, Southwest Hospital, Third Military Medical University, Chongqing 400038, P.R. China; ^3^ Division of Gastroenterology, Department of Medicine, School of Medicine, University of California, San Diego, La Jolla, California 92093, USA

**Keywords:** hepatocellular carcinoma, miR-149, metastasis, PPM1F, microRNA

## Abstract

microRNAs have been implicated in hepatocellular carcinoma (HCC) metastasis, which is predominant cause of high mortality in these patients. Although an increasing body of evidence indicates that miR-149 plays an important role in the growth and metastasis of multiple types of cancers, its role in the progression of HCC remains unknown. Here, we demonstrated that miR-149 was significantly down-regulated in HCC, which was correlated with distant metastasis and TNM stage with statistical significance. A survival analysis showed that decreased miR-149 expression was correlated with a poor prognosis of HCC as well. We found that over-expression of miR-149 suppressed migration and invasion of HCC cells *in vitro*. In addition, we identified PPM1F (protein phosphatase, Mg^2+^/Mn^2+^-dependent, 1F) as a direct target of miR-149 whose expression was negatively correlated with the expression of miR-149 in HCC tissues. The re-expression of PPM1F rescued the miR-149-mediated inhibition of cell migration and invasion. miR-149 regulated formation of stress fibers to inhibit migration, and re-expression of PPM1F reverted the miR-149-mediated loss of stress fibers. Moreover, we demonstrated that over-expression of miR-149 reduced pMLC2, a downstream effector of PPM1F, in MHCC-97H cells. *In vivo* studies confirm inhibition of HCC metastasis by miR-149. Taken together, our findings indicates that miR-149 is a potential prognostic biomarker of HCC and that the miR-149/PPM1F regulatory axis represents a novel therapeutic target for HCC treatment.

## INTRODUCTION

Hepatocellular carcinoma (HCC) is one of the most common malignant tumors and the third leading cause of cancer-related death [1]. Although numerous advanced therapeutic strategies have been utilized in recent years, the 5-year overall survival rate remains very poor due to postsurgical recurrence and metastasis [2]. The high recurrence and metastasis rates of HCC have become the key issue that affects the long-term survival of patients [3]. The pathogenesis of HCC is still not well defined, although many oncogenes or antioncogenes are associated with the metastasis of HCC [4]. Thus, the mechanism of HCC metastasis is urgently needed for HCC therapy.

microRNAs are small non-coding RNAs (21–23 nucleotides) that negatively and post-transcriptionally regulate gene expression, mainly via sequence-specific interactions with the 3′ untranslated regions (UTRs) of cognate mRNA targets [5]. miRNAs play an important role in many cellular processes, such as proliferation, differentiation, apoptosis, and the stress response. Additionally, miRNAs are key regulators in many diseases, including cancers. They function as either oncogene or tumor suppressors and influence the initiation and progression of cancers [6]. Many reports have shown that the deregulation of miRNAs is involved in the growth and metastasis of HCC. For example, up-regulation of miR-182 is related to intrahepatic metastasis and poor prognosis [7], whereas a decrease in miR-124 is significantly associated with a more aggressive phenotype and poor prognosis [8]. miR-149 primarily functions as an anti-tumor miRNA, and its expression is deregulated in multiple types of cancers, including breast cancer [9], colorectal cancer (CRC) [10, 11], gastric cancer [12], head and neck squamous cell carcinoma (HNSCC) [13] and non-small-cell lung cancer (NSCLC) [14]. Chan et al. demonstrated that miR-149 targeted GIT1 to suppress integrin signaling and breast cancer metastasis [9]. Wang et al. reported that Sp1 mediated the link between methylation of tumor suppressor miR-149 and outcome in CRC [10]. However, to date, the mechanism of miR-149 deregulation and its regulatory networks in HCC remain elusive.

Mg^2+^/Mn^2+^-dependent protein phosphatase 1F (PPM1F) belongs to the PP2C family of Ser/Thr protein phosphatases. PP2C family members are known as negative regulators of cell stress response pathways, including p38 MAPK, JNK and HOG signaling pathways. PPM1F is ubiquitous in various tissues and organs. Studies show that PPM1F is involved in the motility and adhesion of cancer cells by regulating cytoskeleton remodeling [15, 16]. For example, Susila et al. reported that the PPM1F level was high in various cancer cell types, and a high expression of PPM1F increased the migration and invasiveness of breast cancer cells [17]. Recent studies have shown that miRNAs could regulate PPM1F. For example, miR-200c targeted PPM1F to suppress invasion, migration, cell polarization, and stress fiber formation in metastatic breast cancer cells by regulating the reorganization of the cytoskeleton [18].

In this study, we demonstrate that miR-149 is frequently down-regulated and significantly correlates with tumor metastasis and poor prognosis in HCC patients. We further proved that miR-149 inhibited the metastasis of HCC *in vivo* and *in vitro* by suppressing actin-regulatory proteins PPM1F.

## RESULTS

### miR-149 is frequently down-regulated in human HCC tissue and associated with poor clinicopathologic features and low postoperative survival rate

**Figure 1:**
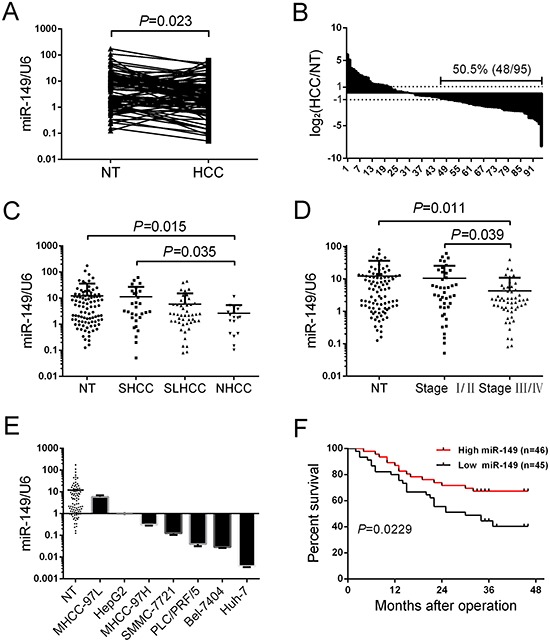
miR-149 is frequently down-regulated in human HCC tissue and associated with poor clinicopathologic features and a low postoperative survival rate **A, B.** The expression of miR-149 in 95 pairs of HCC tissues and their corresponding non-tumorous liver tissues was determined by qRT-PCR. U6 (U6 small nuclear RNA) was used as an internal control. Fold changes were analyzed using the formula 2^−(ΔΔCT[HCC/NT])^. The dotted line indicated a fold change of miR-149 equal to 2. **C.** 95 pairs of HCC tissues and their corresponding non-tumorous liver tissues were divided into the SHCC, NHCC, SLHCC and NT groups. The diagram shows the miR-149 expression of each group. **D.** 95 pairs of HCC tissues and their corresponding non-tumorous liver tissues were divided into three groups, Stage I/II, Stage III/IV and NT. The diagram showed the miR-149 expression of each group. **E.** miR-149 expression in 95 pairs of non-tumorous liver tissues and HCC cell lines. miR-149 expression was lower in HCC cell lines compared with the 95 pairs of non-tumorous liver tissues. Data were the mean ± SD. **F.** Decreased miR-149 expression was significantly associated with the overall survival of 91 HCC patients. The median was used as the cut-off value to divide patients into low and high expression groups. The survival curve was calculated with a Log-rank test.

To determine the expression of miR-149 in HCC, we analyzed 95 cases of HCC tissues and adjacent non-tumorous liver tissues with quantitative real-time PCR. Compared with the adjacent non-tumorous liver tissues, the median level of miR-149 was significantly down-regulated in tumor tissues (*P* = 0.023, Figure 1A). The overall expression level of miR-149 was decreased (more than two-fold [i.e. log_2_ (HCC/NT) < 1]) in 48 HCC samples (50.52%), unchanged in 25 samples (26.32%) and up-regulated in 22 samples (23.15%) (Figure 1B), which indicates that miR-149 is a frequently down-regulated in HCC.

To examine the relationship between miR-149 expression and clinicopathologic features, the patients were divided into two groups according to the median level of miR-149 expression; low miR-149 levels were negatively associated with AFP (*P* = 0.083), distant metastasis (*P* = 0.047), and TNM stage (*P* = 0.017; Table 1) but not with tumor size and histological grade. Based on above clinicopathologic features, miR-149 was related to the metastasis-associated biological parameters of HCC. To better the illustration of role of miR-149 in the metastasis of HCC, the patients were divided into three groups according to their metastatic potential, including solitary large HCC (SLHCC, >5 cm in greatest dimension with 1 solitary tumor node), small HCC (SHCC, tumor diameter ≤5.0 cm) and nodular HCC (NHCC, node number >1). Among the three subtypes, SLHCC and SHCC exhibited the lower invasive and metastatic potential. Conversely, NHCC turned out to be more invasive and metastatic [19, 20]. Our data showed that miR-149 was significantly down-regulated in NHCC compared to SLHCC (Figure 1C). Similarly, we divided the patients into two groups based on TNM stage, and our data showed that miR-149 was more significantly down-regulated in stage III/IV than stage I/II cancers (Figure 1D). Furthermore, the expression level of miR-149 was also significantly reduced in HCC cell lines (all *P* < 0.05; Figure 1E) in comparison to non-tumorous liver tissues (*N* = 95).

**Table 1:** The correlations of miR-149 with clinicopathological features of HCC

Clinicopathologic Variable	*n*	miR-149		*P*
		Low expression	High expression	
Agent				
Female	8	5	3	0.441
Male	87	42	45	
Age(year)				
≤ 60	83	40	43	0.511
> 60	12	7	5	
AFP				
< 20 ng/ml	28	10	18	0.083
≥ 20 ng/ml	67	37	30	
HBsAg				
Negative	6	4	2	0.384
Positive	89	43	46	
Liver cirrhosis				
Absence	64	33	31	0.558
presence	31	14	17	
Tumor size(cm)				
≤ 5	38	16	22	0.241
> 5	57	31	26	
Tumor nodule number				
Solitary	74	36	38	0.763
Multiple(≥2)	21	11	10	
TNM Stage				
I/II	42	15	27	0.017
III/IV	53	32	21	
Histological grade				
Well and moderately	82	42	40	0.393
Poorly	13	5	8	
Distant metastasis				
Absence	68	30	38	0.047
presence	27	18	9	

We next analyzed the relationship between miR-149 expression and the patients' prognoses. The patients (91 cases) were divided into two groups according to the median level of miR-149 expression as either high expression (46 patients) or low expression (45 patients). The survival curves showed that the overall survival of HCC patients with high miR-149 expression was shorter than that of patients with low miR-149 expression (Figure 1F). In conclusion, these results reveal that decreased miR-149 expression correlates with poor HCC prognosis, which implies that miR-149 participates in HCC progression.

### Exogenetic over-expression of miR-149 suppresses HCC cell migration and invasion *in vitro*

**Figure 2:**
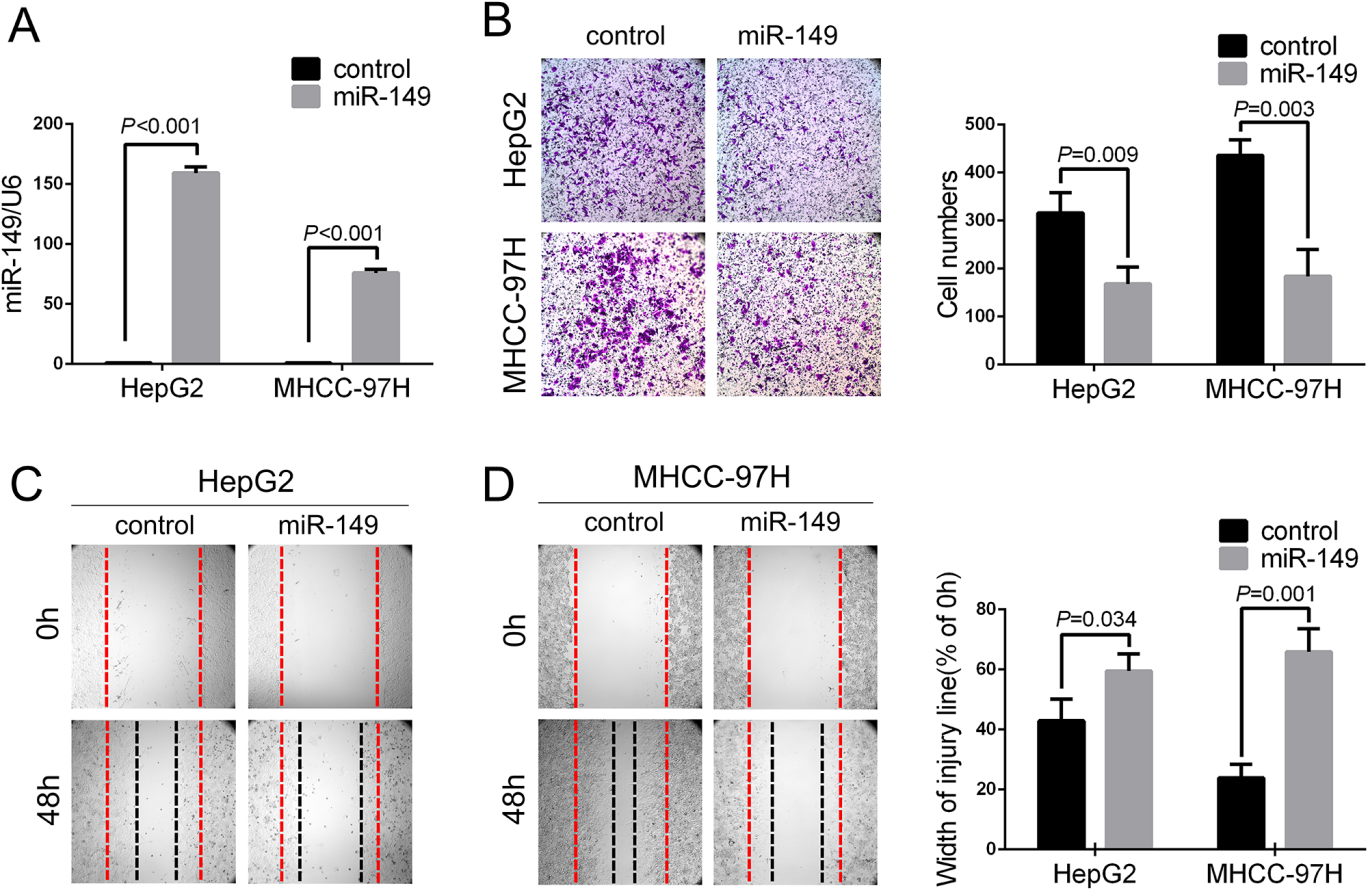
Exogenetic over-expression of miR-149 suppresses HCC cell migration and invasion *in vitro* **A.** The over-expression of miR-149 was confirmed in HepG2 and MHCC-97H cells using qRT-PCR. The ability of cells to invade and migrate was compared with a **B.** Transwell assay and **C, D.** wound healing assay. Representative images (left panel) and the quantification of three randomly selected fields (right panel) are shown. A statistical analysis was performed with Student's *t* test.

In light of the correlation of miR-149 with tumor metastasis in HCC patients, we investigated its role in HCC cells. Among the seven HCC cell lines (Figure 1E), HepG2 and MHCC-97H expressed miR-149 at relatively low levels but were most metastatic. Hence, we selected the HepG2 and MHCC-97H cell lines and transduced them with a miR-149 lentivirus. The transduction efficiency was confirmed with fluorescence image (Supplementary Figure S1) and real-time PCR (*P* < 0.001; Figure 2A). We next investigated the potential role of miR-149 in modulating the ability of HCC cells to invade and migrate. The results of Transwell assays with matrigel revealed that HepG2 and MHCC-97H cells overexpressing the miR-149 lentivirus exhibited significant reduction in rates of invasion compared to control cells (Figure 2B). Similarly, wound-healing assays indicated that the over-expression of miR-149 slowed wound healing in HepG2 and MHCC-97H cells (Figure 2C, 2D). In addition, the effects of miR-149 on the proliferation capacities of HCC cells were evaluated with cck8 assays, indicating miR-149 did not markedly influence the proliferation of HepG2 and MHCC-97H cells (data not shown).

### Actin-regulatory proteins PPM1F are direct target of miR-149

**Figure 3:**
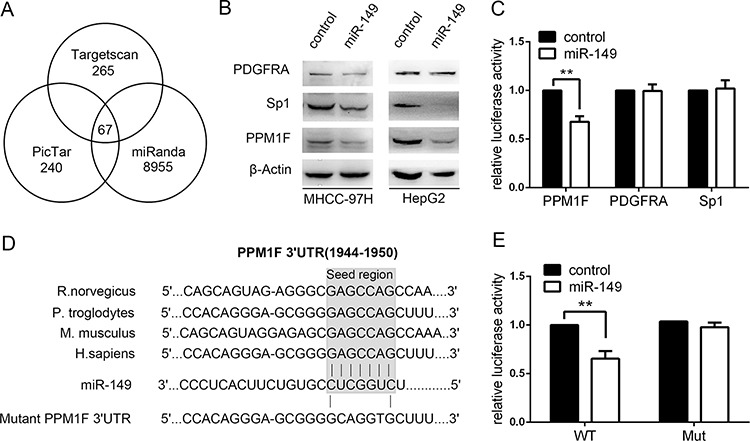
miR-149 down-regulates PPM1F expression by directly targeting its 3′-UTR **A.** The candidate genes were identified with a bioinformatics prediction of putative target genes using TargetScan, PicTar and miRanda. **B.** The protein levels of PPM1F in MHCC-97H and HepG2 cells were determined by western blot assays after treating cells with miR-149 or a negative control lentivirus. β-actin served as an internal control. **C.** Relative luciferase activity assays for luciferase reporters with wild-type PPM1F, Sp1, PDGFRA 3′-UTR were performed after co-transfection with pMIR–miR-149 plasmids or a vector control. The relative luciferase activities were analyzed in HEK-293T cells. The Renilla luciferase vector was co-transfected as an internal control. **D.** The sketch of putative binding sites, corresponding mutant sites of PPM1F 3′-UTR, and the interspecies conservation of seed matching sequences were marked by a gray box. **E.** The Luciferase assay with wild-type PPM1F or mutant 3′-UTR was carried out as described previously. *p < 0.05; **p < 0.01.

To disclose the mechanism by which miR-149 affected cell migration and invasion in HCC, we attempted to identify the potential target genes of miR-149. Three databases, TargetScan, PicTar and miRanda, were searched for potential target genes under the control of miR-149. The interactions of 67 targets were predicted using the search programs (Figure 3A). Among these predicted targets, 6 candidate genes were selected for further validation due to their metastatic properties. Next, the protein levels of these 6 candidate genes were measured. After the preliminary screen, we identified PPM1F (protein phosphatase, Mg2+/Mn2+ dependent, 1F), SP1 and PDGFRA as possible targets of miR-149 (Figure 3B). Hereby, we constructed vectors by directly fusing the 3′-UTR of PPM1F, SP1 and PDGFRA and the binding site of miR-149 downstream of the luciferase reporter gene. The vector was co-transfected with miR-149 or a control into HEK293T cells, and the relative luciferase activity was detected. The results show that miR-149 significantly decreased the relative luciferase activity in the 3′-UTR of the PPM1F vector compared with the control, whereas the luciferase activity was not altered in SP1 and PDGFRA (Figure 3C). To further verify this result, a point mutation in the tentative miR-149-binding seed region of PPM1F 3′-UTR was constructed (Figure 3D). As expected, point mutations abrogated the suppressive effect of miR-149 on PPM1F (Figure 3E). To conclude, our data indicate that miR-149 negatively regulates the expression of PPM1F by directly targeting its 3′-UTR.

### PPM1F is frequently up-regulated and inversely associated with the expression of miR-149 in HCC

**Figure 4:**
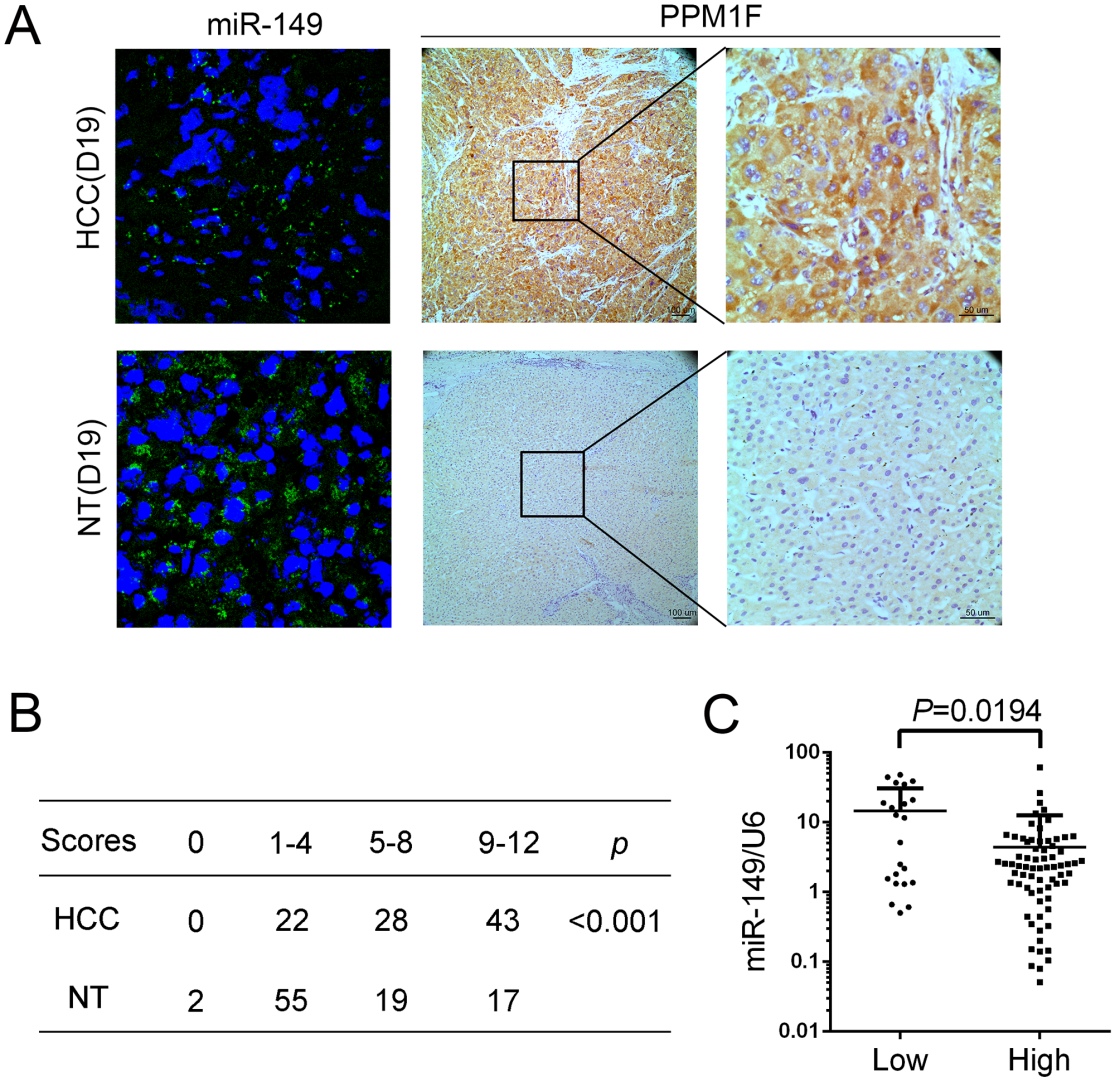
PPM1F is frequently up-regulated and inversely associated with the expression of miR-149 in HCC **A.** Detection of miR-149 (Fluorescence *in situ* hybridization) and PPM1F (Immunohistochemistry) expression in the same HCC and corresponding non-tumorous tissue (D19). The cell nuclei were stained with DAPI (blue). **B.** The statistical analysis of 95 pairs of HCC tissues and their corresponding non-tumorous liver tissues according to immunohistochemical staining scoring. Statistical significance was assessed using the X2 test. **C.** Correlation analysis between the expression levels of miR-149 and PPM1F. The scatter plot indicated the relative expression of miR-149 in the high or low PPM1F expression groups. The expression levels of PPM1F were ranked according to the results of immunohistochemical staining. Scores of 1 or 2 were classified as low expression, and scores of 3 or 4 were classified as high expression. A statistical analysis was performed using Student's *t* test.

PPM1F is reportedly up-regulated in various cancer cell types and involved in breast cancer metastasis. However, the expression of PPM1F in HCC was unknown. The protein level of PPM1F was examined with IHC in 93 pairs of paraffin-embedded HCC tissues and corresponding non-tumorous liver tissues. The data show that PPM1F was mainly expressed in the cytoplasm of cells, and this expression was increased in HCC tissues compared with the corresponding non-tumorous liver tissues (Figure 4A). Of the 93 HCC specimens, PPM1F staining was strong (score of 5–8 or 9–12) in 71 specimens (76.4%) and weak (score of 0, or 1–4) in 22 specimens (23.6%) (Figure 4B). In contrast, PPM1F staining was strong (score of 5–8 or 9–12) in 36 (38.7%) and weak (score of 0, or 1–4) in 57 (61.3%) corresponding non-tumorous liver tissues specimens. Furthermore, to determine whether the high expressed PPM1F is related to miR-149, we divided the HCC tissues into high (score of 5–8 or 9–12) and low (score of 0, or 1–4) PPM1F expression groups. We compared the miR-149 expression in both groups, and showed that miR-149 levels are inversely correlated to PPM1F levels in HCC samples (Figure 4C). To further validate the inverse relationship between PPM1F and miR-149, we examined miR149 and PPM1F levels in the same HCC tissues. Fluorescence *in situ* hybridization results showed that the miR-149 expression in HCC tissues was lower than their corresponding non-tumorous liver tissues, as opposed to PPM1F levels which were higher in HCC tissues compared to the non-tumorous controls (Figure 4A).

### miR-149 exerted its function by suppressing PPM1F expression

**Figure 5:**
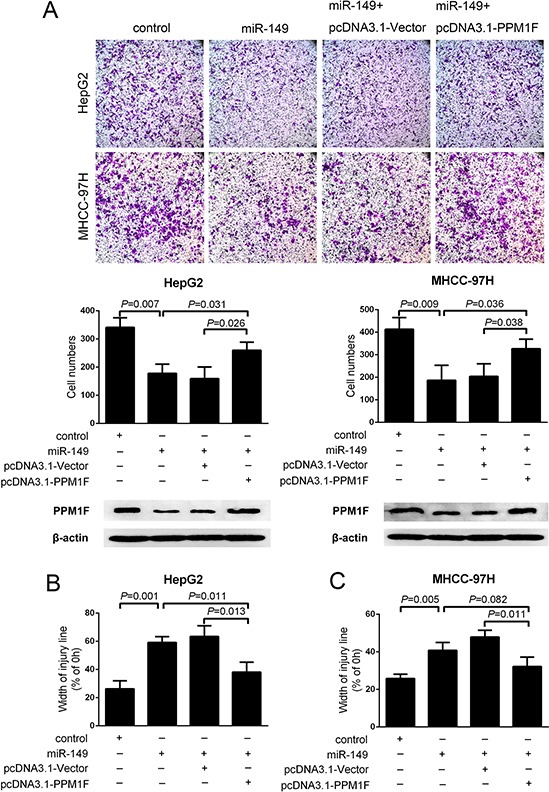
PPM1F rescued the effect of miR-149 on the invasion and migration of HCC cells **A.** Transwell invasion assays and **B, C.** wound healing assays were performed in of MHCC-97H and HepG2 cells after the reintroduction of PPM1F (lack the 3′-UTR) or control plasmid. PPM1F protein levels were detected by western blot analysis. A statistical analysis was performed by using Student's *t* test.

To examine whether miR-149 exerted its function via PPM1F, we first constructed a pcDNA3.1-PPM1F plasmid that contains the coding sequence but lacks the PPM1F 3′UTR and then transfected it into HepG2 and MHCC-97H cells which stably transduce the control and miR-149 lentivirus. Western blotting results indicated the successful PPM1F transfection. Transwell assays showed that the reintroduction of PPM1F abrogated the suppression of cell invasion induced by miR-149 in both HepG2 and MHCC-97H cells (Figure 5A, Supplementary Figure S2A, S2B). Similarly, the wound healing assay also confirmed that the reintroduction of PPM1F rescued the effect of miR-149 on the migration of HCC cells (Figure 5B, 5C).

**Figure 6:**
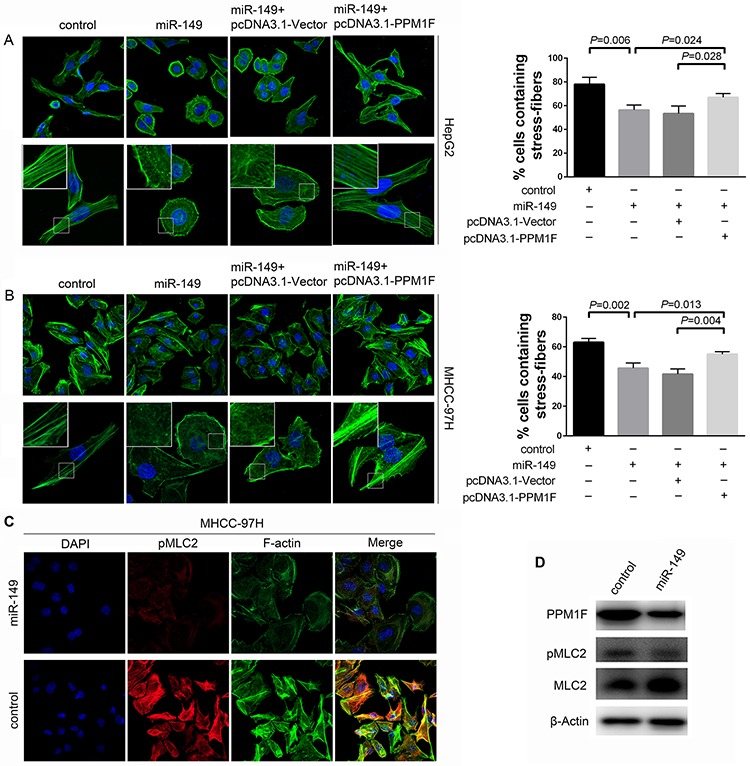
miR-149 regulated stress fiber formation, and PPM1F rescued the loss of stress fibers in HCC cells **A, B.** Immunofluorescent staining of stress fibers in HCC cells. MHCC-97H and HepG2 cells were transduced with miR-149 or negative control lentivirus, and transfection with pcDNA3.1-Vector or pcDNA3.1-PPM1F plasmid. Stress fibers were stained with Alexa Fluor 488-phalloidin, and cell nuclei were stained with DAPI. The percentage of stress fiber-containing cells was determined by counting 100 to 200 cells per experiment. Representative images (left panel) and data show the quantification of three independent experiments (right panel). **C.** The over-expression of miR-149 reduced the level of pMLC2 in MHCC-97H cells. Cells were transduced with miR-149 or negative control lentivirus, and cells were stained for F-actin with Alexa Fluor 488-phalloidin (green), pMLC2 with antibody (red), and nuclei with DAPI (blue). **D.** The levels of PPM1F, pMLC2 and total MLC2 were detected by Western blotting in MHCC-97H cells that over-expressed miR-149 or control cells. β-actin served as an internal control.

It is reported that PPM1F promote metastasis by influencing the cytoskeletal reorganization in other cancers [16, 21, 22]. Thus, we firstly investigated the effect of miR-149 on the formation of stress fibers in HCC. Interestingly, immunofluorescence staining showed a dramatic loss of stress fibers with a concomitant relocation of actin filaments to the cell periphery in HepG2 cells due to the over-expression of miR-149 (Figure 6A). Approximately 56% of cells over-expressed miR-149 contained stress fibers, as opposed to 75–80% of control cells. To further confirm whether PPM1F effects on the stress fiber reduction caused by miR-149 over-expression. We re-introduced PPM1F to HepG2 cells that over-expressed miR-149, resulting in abrogation the loss of stress fibers. Then, we validated these findings in MHCC-97H cells. The over-expression of miR-149 in the cells significantly reduced the formation of stress fibers by approximately 15–20% (Figure 6B). Moreover, the re-introduction of PPM1F to MHCC-97H cells rescued the inhibition of stress fiber formation. Taken together, these data indicated that miR-149 suppressed HCC cell invasion and metastasis by suppressing PPM1F which mediates the formation of stress fibers.

The phosphorylation of myosin light chain 2 (pMLC2) at Thr18 and Ser19 is a reported key process in stress fiber formation because it facilitates the assembly of myosin into bipolar filaments and allows stress fibers to contract during migration [23]. Studies have shown that PPM1F could affect MLC2 phosphorylation in breast cancer [18]. To explore whether the molecular mechanism is also present in HCC, we examined the expression of pMLC2 by immunofluorescence. Our data showed miR-149 over-expression induced significant reduction in co-localization of pMLC2 with actin fibers compared to control in MHCC-97H cells (Figure 6C). The western blotting data were consistent with the immunofluorescence data (Figure 6D). However, the expression of pMLC2 showed no obvious difference in HepG2 cells (data not shown), and we speculated that other mechanism may be involved in this process.

### Ectopic miR-149 expression inhibits HCC cell metastasis *in vivo*

**Figure 7:**
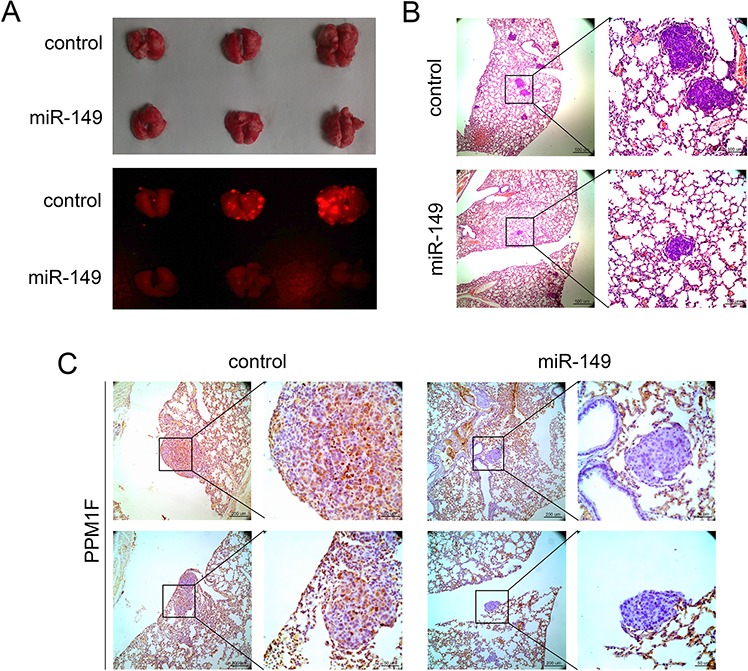
miR-149 suppresses HCC metastasis *in vivo* **A.** The effects of miR-149 on HCC metastasis. HepG2 cells transduced with miR-149 expression lentivirus or negative control lentivirus were used to generate the mouse model. Images show the fluorescence of lung metastases photographed with a whole-body fluorescent imaging system. **B.** Hematoxylin and eosin staining was performed to identify HCC lung metastases, and representative sections of lung metastases are shown. The number of lung metastatic nodules distinctly decreased in the miR-149 expression group. **C.** Immunohistochemical staining of lung metastasis sections was performed to analyze the expression of PPM1F. Representative images show that the expression of PPM1F decreased in the miR-149 expression group.

We further confirmed the inhibition of HCC by miR-149 *in vivo*. A metastatic HCC model was used to confirm the effect of miR-149 on HCC metastasis. RFP-miR-149-HepG2 or RFP-vector-HepG2 cells were injected into the caudal veins of nude mice to induce pulmonary metastasis. The mice were sacrificed 6 weeks later, and the lungs were removed and observed with a whole-body fluorescent imaging system. As shown in Figure 7A, the over-expression of miR-149 reduced the fluorescence signal due to lung metastasis compared with the control group. Consistently, the H&E staining of tumor sections showed that the incidence of pulmonary metastasis decreased in the miR-149 group (Figure 7B). Moreover, the immunohistochemistry of PPM1F in the transplanted tumors was clearly down-regulated in the miR-149 over-expression group compared with the control group (Figure 7C). Hence, these observations suggest that miR-149 may suppress HCC metastasis by down-regulating PPM1F *in vivo*.

## DISCUSSION

Accumulated studies have shown that expression of microRNA is deregulated in human cancers, and the role of this regulation during oncogenesis has been highlighted. Several studies have shed light on the role of miRNA in treatment and as a prognostic indicator. For example, a robust 7-miRNA signature in gastric cancer can predict overall survival and relapse-free survival [24]. Similarly, low levels of miR-26 in HCC are an independent predictor of poor survival [25]. miR-149 also predicts overall survival in patients suffering from HNSCC [13] and CRC [10]. In the present study, we demonstrated that miR-149 was frequently down-regulated in HCC tissues compared to non-tumorous liver tissues, and the level of miR-149 is positively correlated with survival rate. Our prospective study indicated miR-149 may serve as a potential prognostic biomarker in clinical practice.

Invasion and metastasis are two of the most important hallmarks of malignancy and major causes of cancer-related death. Previous studies have shown that miRNAs are associated with these two events in HCC. miR-17-5p inhibited HCC cell invasion and metastasis by regulating the AKT/mTOR pathway [26]. miR-331-3p promoted proliferation and metastasis of HCC by targeting PHLPP [27]. Furthermore, miR-149 can reportedly regulate invasion and migration in various types of malignant tumors. For example, miR-149 suppresses metastasis of breast cancer via directly targeting GIT1 expression which compromises integrin signaling [14]. In glioblastoma, miR-149 inhibited proliferation and invasion of glioma cells by blocking AKT1 signaling [28]. However, the function of miR-149 in HCC has not been reported previously. In the present study, we demonstrated that ectopic miR-149 expression markedly repressed HCC invasion and migration *in vitro* and *in vivo*. Therefore, our results suggested that miR-149 is a novel potential therapeutic target for HCC treatment.

To further elucidate the underlying molecular mechanism by which miR-149 is involved in HCC metastasis, we predicted the putative targets of miR-149 using bioinformatics and validated that PPM1F is a direct downstream mediator of miR-149 through luciferase reporter and western blot assays. PPM1F is reportedly up-regulated in various cancer cell types, including cervical cancer, gastric cancer, breast cancer and neuroblastoma, compared with cell lines that are derived from normal tissues [17]. However, the role of PPM1F in HCC remained unknown. The immunohistochemical staining in this study demonstrated that the expression of PPM1F was significantly up-regulated in HCC patient specimens. Furthermore, we found that the expression of PPM1F was inversely correlated with the expression of miR-149 in HCC, demonstrating that down-regulation of PPM1F is, at least in part, caused by up-regulation of miR-149 in HCC. To testify that PPM1F is a major downstream effector of miR-149, we used a gain-of-function approach to functionally characterize PPM1F in cellular invasion and migration assays. Our results showed that over-expression of PPM1F antagonized the functions of miR-149, which indicates that PPM1F is the primary functional target of miR-149 in HCC. However, the data also showed that the over-expression of PPM1F could not completely reverse the miR-149-mediated inhibition of invasion and migration in both HepG2 and MHCC-97H cells, which suggests that miR-149 may target other factors in HCC. Furthermore we analyzed the effect of miR-149 overexpression in two HCC cell lines, HepG2 and MHCC97H, on the expression of PPM1F and other putative target genes (GIT1, SP1, FOXM1) previously reported by other groups to promote migration and invasion in other cancer cell lines [9, 10, 14]. We observed that overexpression of miR-149 caused a significant decrease in the expression of PPM1F in both HCC cell lines. However, the effect of miR-149 on the other putative target genes was not consistent (Supplementary Figure S3). This suggests that there are other cell-type specific factors involved in the regulation of SP1, GIT1, and FOXM1, while miR-149 seems to be the primary negative regulator of PPM1F in HCC cells. Therefore, our data provided the first evidence that PPM1F is directly regulated by miR-149 and plays an important role in HCC metastasis.

Our study has shown that PPM1F could promote HCC metastasis, but the mechanism associated with this promotion is unclear. miR-200c can reportedly directly target PPM1F and repress breast cancer cell invasion and migration by modulating the actin cytoskeleton reorganization during EMT [18]. Susila et al. reported that PPM1F knockdown causes a loss of stress fibers and prominent focal adhesions in breast cancer cells [17]. Xie and colleagues reported that PPM1F prevented stress fiber loss by interacting with the formin protein mDia1 [16]. Previous studies suggest that PPM1F can promote the formation of stress fibers. Moreover, studies have shown that stress fibers play an important role in HCC metastasis. For example, Ma et al. reported that the knockdown of RhoE enhanced the phosphorylation of myosin phosphatase, promoted the assembly of stress fibers, and enhanced the local invasion of HCC [19]. Tang and colleagues reported that the over-expression of eukaryotic initiation factor 5A2 enhanced cell motility and promoted tumor metastasis by activating RhoA/Rac1 to stimulate the formation of stress fibers and lamellipodia in HCC [30]. Therefore, we detected the amount of stress fibers in control and miR-149-over-expressing HCC cells to investigate whether the inhibition of HCC progression by miR-149 mediated by PPM1F was related to the formation of stress fibers. miR-149 was involved in the formation of stress fibers by targeting PPM1F in both HepG2 and MHCC-97H cells. Our study proposes a new mechanism: the over-expression of miR-149 directly decreases PPM1F expression, inducing cytoskeletal remodeling that causes the loss of stress fibers and suppression of HCC cell migration and invasion.

Next, we explored the mechanism by which PPM1F can promote the formation of stress fibers and affect HCC metastasis. The contractile activity of stress fibers can be observed during HCC migration, and pMLC2 is known to play an important role in this process by facilitating the assembly of myosin into bipolar filaments. Myosin filaments have been shown to contribute to stress fiber formation by cross-linking actin filaments [23]. In addition, PPM1F reportedly increases the phosphorylation of MLC2, which promotes breast cancer metastasis [18]. Thus, we sought to determine whether the targeting of PPM1F by miR-149 decreases the phosphorylation of MLC2 in HCC cells. Our results showed that the level of pMLC2 was decreased in MHCC-97H but not HepG2 cells with over-expressed miR-149, which suggested that another mechanism could be involved in this process. In fact, studies have reported the mechanism by which PPM1F promotes the formation of stress fibers. Rho GTPases are known to act as molecular switches regulating diverse cellular signaling pathways that are involved in the modulation of the actin cytoskeleton [31]. Ras homolog gene family, member A (RhoA) acts on Rho kinase (ROK, ROCK) and formin protein mDia to modulate stress fibers [32]. PPM1F can reportedly interact with mDia1, and the interaction between mDia1 and PPM1F can regulate the actin cytoskeleton [16]. Another study reported that PPM1F bound to the guanine nucleotide exchange factor PIX [22], and PIX has been shown to interact with p21(Cdc42/Rac)-activated kinase (PAK) [33]. Thus, the combination of POPX, PAK and PIX dephosphorylated and down-regulated PAK, which blocked the phenotypic promotion of the loss of stress fibers by PAK [22]. These studies provided clues for the potential mechanisms in HepG2 cells by which miR-149 targets PPM1F to impair the formation of stress fibers, but the mechanism requires further investigation.

In conclusion, miR-149 is expressed at low levels in HCC and is significantly correlated with poor patient prognosis. Our data indicated that miR-149 suppressed the invasion and migration of HCC by directly targeting PPM1F. A decrease in the expression of PPM1F leaded to a loss of stress fibers by decreasing the phosphorylation of MLC2. The newly identified miR-149/PPM1F axis provided new insight into the pathogenesis of HCC and a novel potential therapeutic target for the treatment of HCC.

## MATERIALS AND METHODS

### Patients and tissues specimens

From January 2010 to December 2012, a total of 95 pairs of HCC tissues and adjacent non-tumorous liver tissues were gathered from patients who underwent liver resection at the Department of Surgery, Southwest Hospital of The Third Military Medical University. The clinical and pathological features of these patients are described in Table 1. The histopathology was evaluated by two certified pathologists at the Department of Pathology at Southwest Hospital of The Third Military Medical University. The use of human tissues was approved by the Institutional Review Board of the Third Military Medical University.

### Quantitative real-time PCR (qRT-PCR)

The Trizol Reagent (TaKaRa) was used to extract the total RNA according to the manufacturer's instructions. For miRNA level detection, the complimentary DNA was synthesized from 2 ng of total RNA with the Prime Script RT reagent Kit (TaKaRa). The qRT-PCR analysis was performed in StepOnePlus™ Real-Time PCR system (applied biosystems) by using SYBR Green qRT-PCR master mix (TaKaRa). The relative expression ratio of miR-149 in each paired tumor and non-tumorous tissue was calculated with the 2^− ΔΔCT^ method; the expression level of snRNA U6 was used as a reference gene. For mRNA level detection, the method is basically same as miRNA detection except using GAPDH as internal control. Primers are described in the Supplementary Table S1.

### Cell culture

The following cell lines were purchased from the American Type Culture Collection (ATCC, Manassas, VA): HepG2, PLC/RFP/5, Bel-7404, Huh-7 and SMMC-7721. The MHCC-97H and MHCC-97L cells were obtained from the Type Culture Collection of the Chinese Academy of Sciences (Shanghai, China). All cells were grown in DMEM medium (Invitrogen, Carlsbad, CA, USA) supplemented with 10% fetal bovine serum (HyClone, Logan, Utah, USA) and 1% penicillin/streptomycin. The transfection and starvation media were free of penicillin-streptomycin and FBS, respectively.

### Wound-healing assay

Cells (5 × 10^5^/well) were transduced with miR-149 or control lentivirus and seeded in six-well plates, cultured overnight and transfected with PPM1F or negative control plasmid. After forty-eight hours, a 500-mm-wide scratch was made across the cells using a sterile plastic tip, and the cells were washed twice with culture medium. Fresh serum-free medium was then added, and the imaging was performed after 0 h and 48 h using an OLYMPUS IX81 microscope and a Retiga-4000DC camera. The images were analyzed using Cell Profiler.

### Invasion assay

An invasion assay was conducted as previously described [34]. The cells were transduced with lentivirus as described above and seeded into chambers with serum-free medium (to monitor cell growth at lowest speed). The images were acquired after 24 h using an OLYMPUS IX81 microscope and a Retiga-4000DC camera. The number of invasive cells from five random areas of the membrane was counted with CellProfiler.

### Western blotting

The protein lysates were prepared and Western blotting was conducted as previously described [35]. All antibodies (see Supplementary Table S2) were diluted in 5% milk/TBST, except for anti-pMLC2, which was diluted in 5% BSA/TBST. The signals were detected with the ECL Western blotting analysis system (Amersham Biosciences, Piscataway, NJ, USA).

### Vector construction and luciferase reporter assay

To test whether miR-149 regulates the expression of the human PPM1F by directly targeting its 3′UTR, we amplified the entire 3′-UTR of human PPM1F using PCR primers (forward primer 5′-CCAGCACCCCAGAGCCAGTCG-3′ and reverse primer 5′-CCCGGAGGTTGGAGGCTGAAG-3′), the products of PCR were inserted into the DraI and SalI restriction sites of the p-MIR-reporter plasmid (Applied Biosystems, Foster City, CA, USA); the insertion was confirmed by sequencing. The pMIR reporter vectors containing mutated versions of the PPM1F 3′ UTR were generated from the wild-type construct with the QuikChange Site-Directed Mutagenesis Kit (Stratagene, La Jolla, CA, USA). The pcDNA 3.1 vector containing PPM1F was purchased from GenePharma (Shanghai, China) and used for the “rescue” experiments.

### Immunofluorescence staining and microscopy

Cells were cultured on circular coverslips in six-well plates, then washed and fixed with 3.7% paraformaldehyde solution for 10 min. The cells were permeabilized with 0.2% Triton X in phosphate-buffered saline (PBS) for 5 min and blocked for 30 min with 3% bovine serum albumin (BSA)-PBS. The cells were then incubated with primary antibodies against pMLC2 (1:200; Cell Signaling Technology) overnight at 4°C and subsequently incubated with Alexa Fluor 647-labeled secondary antibody (1:200; Invitrogen) for 1 h at room temperature. To stain the F-actin cytoskeleton, the cells were incubated with Alexa Fluor 488-phalloidin (1:40; Invitrogen) overnight at 4°C. To visualize the nuclei, the cells were counterstained with DAPI (Sigma) for 10 min. All antibodies and staining reagents were diluted in 3% BSA-PBS, and the cells were washed with PBS three times after each step. The cells were then mounted on glass slides with glycerin, and pictures were taken with a Zeiss LSM 700 Meta confocal microscope.

### Histopathology and immunohistochemistry

Pulmonary tissues from the nude mice were fixed in 10% formalin and embedded in paraffin. For the histopathologic analysis, the tissue sections were stained with hematoxylin and eosin (H&E). The remaining tissue sections were used for immunohistochemistry. The sections were incubated with anti-human PPM1F (1:100, Abcam) overnight at 4°C, followed by incubation with biotinylated secondary antibodies. The staining was visualized using diaminobenzidine. The pathology of HCC tissues and non-tumorous liver tissues slides was examined by a pathologist, and then the slides were subjected immunohistochemistry using antibodies as described above. Representative photos were taken with an OLYMPUS IX81 microscope and a Retiga-4000DC camera.

### Immunohistochemistry evaluation

Semi-quantitative immunohistochemistry detection was used to determine the PPM1F protein levels. The positive reaction was scored into 4 grades according to the intensity of staining: 0, 1, 2 and 3. The percentages of positively stained cells ware scored into 5 grades: 0 (0%), 1 (1–25%), 2 (26–50%), 3 (51–75%) and 4 (76–100%). The product between intensity and percentage scores was used as a final IHC staining score. For the purpose of statistical analysis, the HCC samples was grouped into the low expression (score 0, scores 1–4) and high expression (scores 5–8, scores 9–12) groups.

### Fluorescence *in situ* hybridization

The expression of miR-149 in HCC tissues was detected by fluorescence *in situ* hybridization. The human miR-149 mature sequence is: UCUGGCUCCGUGUCUUCACUCCC. We used (LNA)-based probes to direct against the full length mature miRNA sequence. The 5′-FAM labelled miR-149 probe sequence is: gGGAGTGAAGACACGGAGCCAgA, purchased from BioSense (Guangzhou, China). The fluorescence *in situ* hybridization procedure was followed as the BioSense instructions. Briefly, frozen sections were fixed with 4% paraformaldehyde solution for 30 min, then washed with PBS twice. Fixed slides were then treated with proteinase K at 37°C for 10 min, followed by dehydration in 70%、85%、and 100% ethanol for 5 min. The probe was then added to the slides, which were denatured at 78°C for 5 min. Hybridization was then carried out overnight at 42°C in a humid chamber. The next day, post-hybridization washes were performed in 50% formamide / 2 x SSC at 43°C and followed by 2 x SSC at room temperature to remove non-specific and repetitive RNA hybridization. Finally, slides were counterstained with DAPI (Sigma) for 10 min and examined with a Zeiss LSM 700 Meta confocal microscope.

### *In vivo* metastasis assay

Female BALB/c nude mice (4–5 weeks) were used for the animal studies and housed under standard conditions. The animals were divided into two groups (5 per group) to assess tumor metastasis *in vivo*. One of the groups received intravenous injections of cells that stably over-expressed miR-149, and while the group was injected with control cells (5 × 10^6^ in 0.2 ml PBS) via the lateral tail vein. Six weeks later, the mice were sacrificed, and the lung metastases were visualized with an *In-Vivo* Imaging System (Cambridge Research & Instrumentation, MA, USA). The lungs were excised, fixed with formaldehyde solution and embedded in paraffin. The paraffin fixed tissues were sectioned and stained with hematoxylin-eosin to identify metastatic nodules, and the remaining tissue sections were used for immunohistochemistry. The experiments were performed in accordance with the NIH Animal Use Guidelines and a protocol approved by the responsible authorities.

### Statistical analysis

All data are presented as the means ± S.D of at least three independent experiments and were analyzed with the Prism 6.0 software (GraphPad, San Diego, CA, USA). The paired samples *t*-test was used to assess differences in the miRNA expression levels between HCC tissues and non-tumorous liver tissues. An independent samples *t*-test was used for two groups, while a one-way ANOVA was used for three or more groups. The Kaplan–Meier method was used to analyze survival. *P* values less than 0.05 were considered statistically significant.

## SUPPLEMENTARY FIGURES AND TABLES


