# Supplementary material for: miR-149 represses metastasis of hepatocellular carcinoma by targeting actin-regulatory proteins PPM1F
Source: Oncotarget. 2015 Oct 14;6(35):37808–23. doi: 10.18632/oncotarget.5676 (PMC4741967; doi:10.18632/oncotarget.5676)
Supplement: Supplementary file 1 [file oncotarget-06-37808-s001.pdf]

SUPPLEMENTARY FIGURES AND TABLES

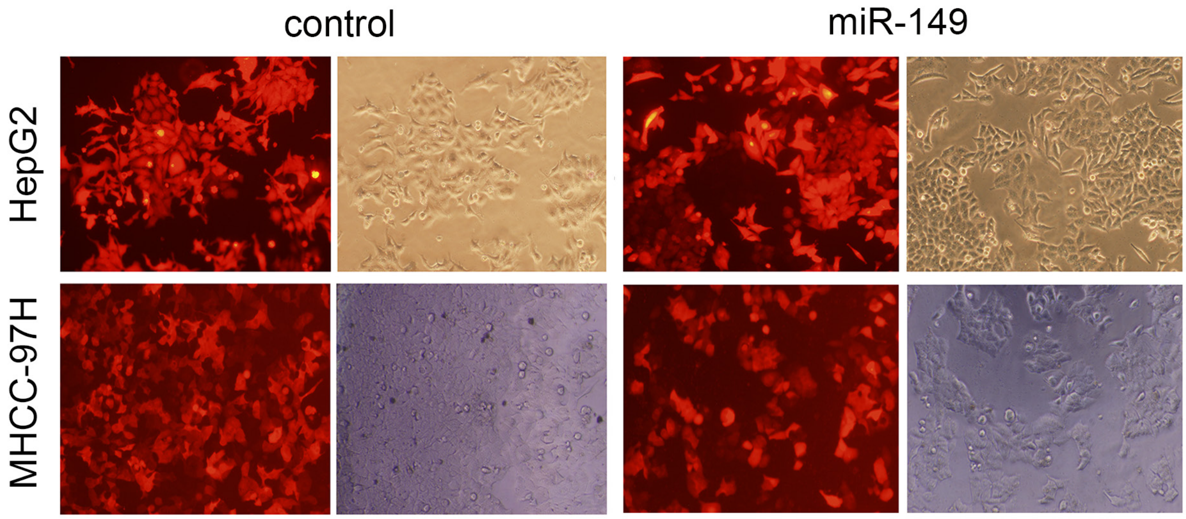

Supplementary Figure S1: HepG2 and MHCC-97H cells transduced with miR-149 over-expression or negative control lentivirus were used in the study; the lentivirus was labeled with red fluorescence protein (RFP).

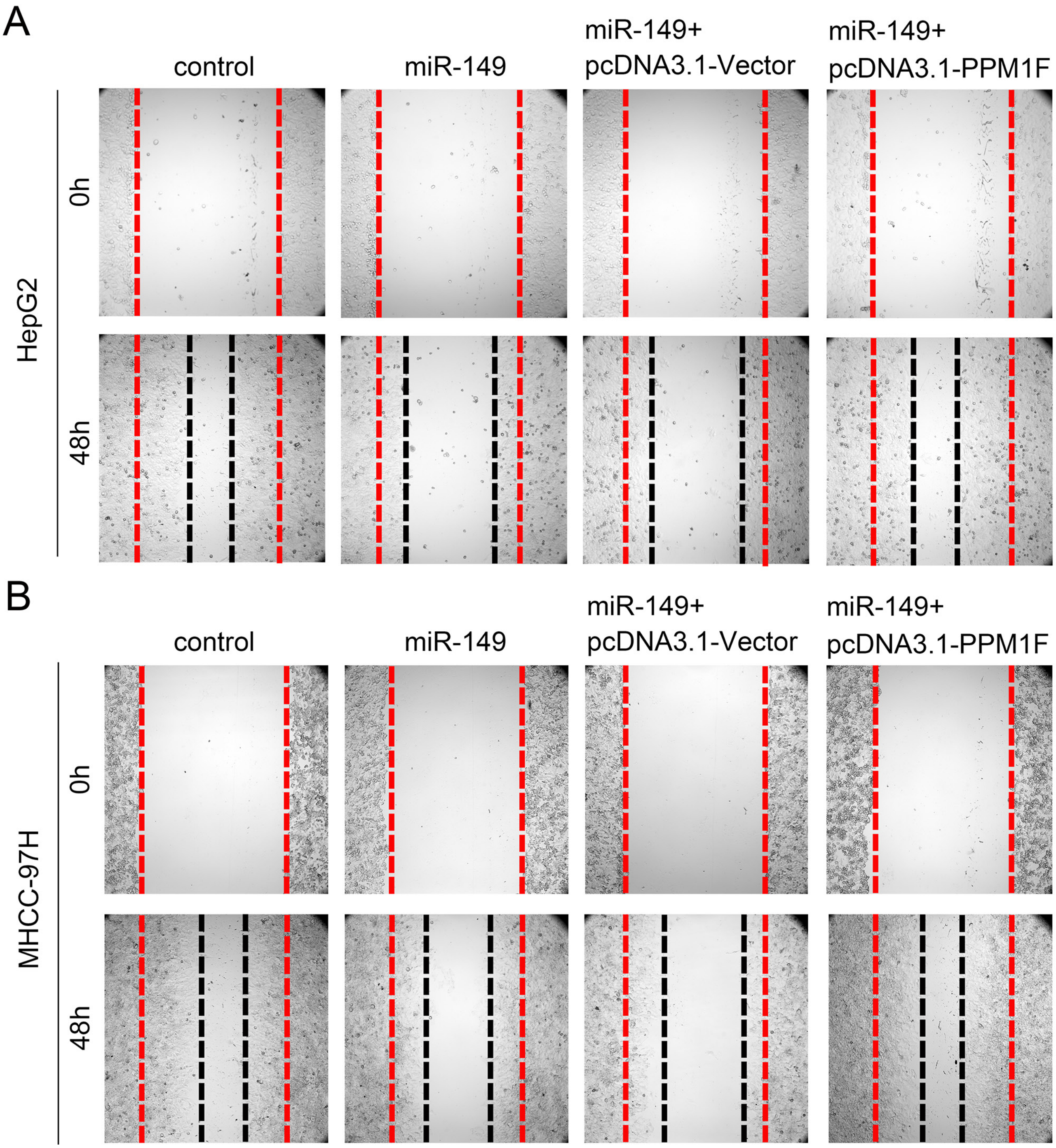

Supplementary Figure S2: Wound healing assay was performed in HepG2 A. and MHCC-97H B. cells after transduction with miR-149 or negative control lentivirus, and transfection with pcDNA3.1-Vector or pcDNA3.1-PPM1F plasmid.

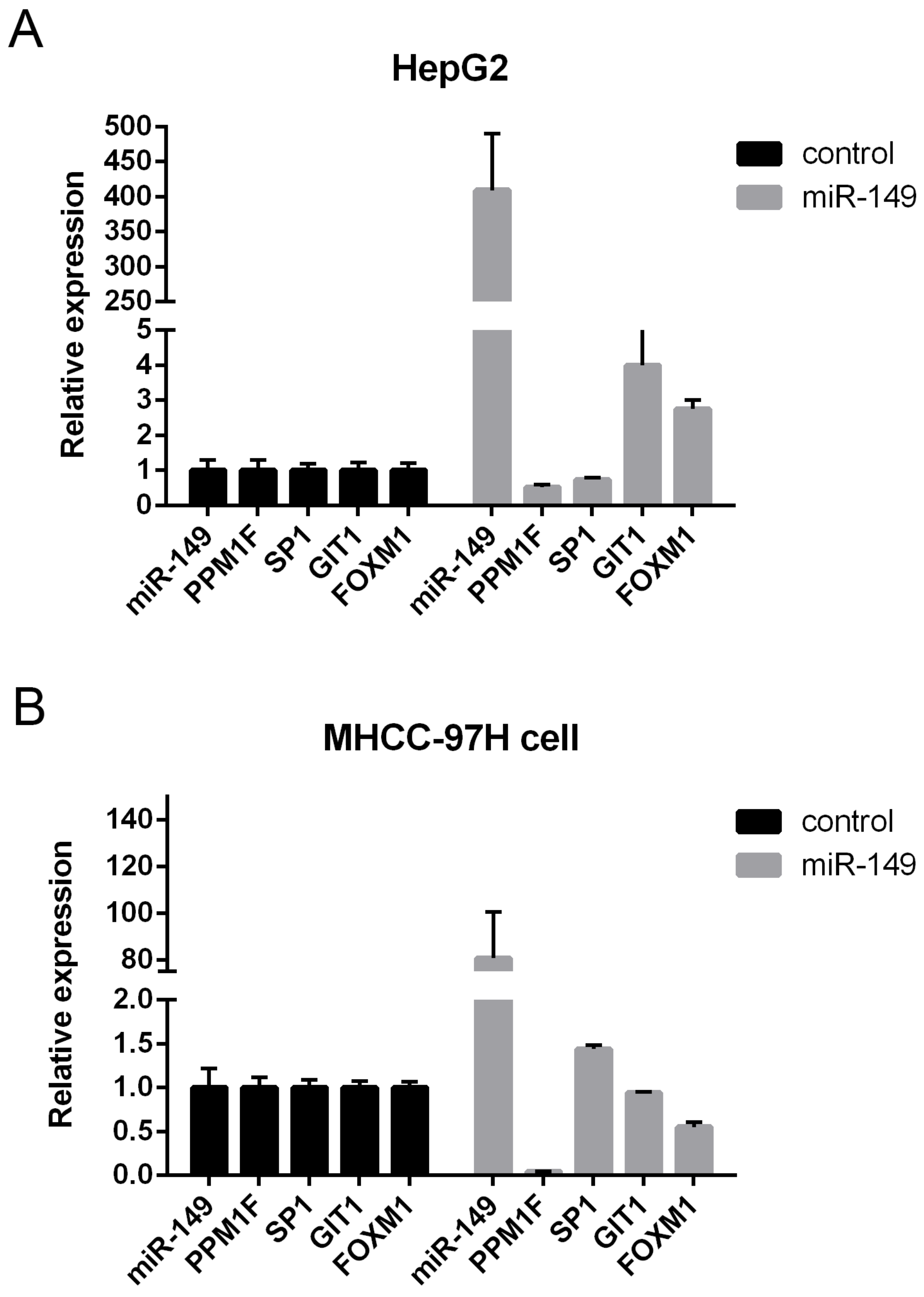

Supplementary Figure S3: qRT-PCR was performed in HepG2 A. and MHCC-97H B. cells to analyze the effect of miR-149 overexpression on the expression of PPM1F and other putative target genes (SP1, GIT1, FOXM1).

Supplementary Table S1: Sequences of primers used for qRT-PCR

Transcript	Sequence	Size
PPM1F	5′-GGAGGAGGACGATGACGAGGAG-3′	82 bp
	5′-GCGGTTAAAGAAACTCTGTGCCAG-3′	
SP1	5′-ATTGGGTACTTCAGGAATCCAGGTG-3′	115 bp
	5′-TCCATCACCACCAGCCCCAT-3′	
GIT1	5′-TAAAGCCAACCCCCAAGACAAAG-3′	116 bp
	5‘-CGGTGACTCCATCATCGTCCC-3‘	
FOXM1	5′-CAAAAGGACAGAAGTGACCCTGGAG-3′	116 bp
	5′-CACCATCACAGGTCTCCCGTTTC-3′	
GAPDH	5′-CATGAGAAGTATGACAACAGCCT-3′	113 bp
	5′-AGTCCTTCCACGATACCAAAGT-3′	
miR-149	5′-ATTCTCTCATCTGGCTCCGTGT-3′	72 bp
	5′-TATGGTTGTTCTGCTCTCTGTGTC-3′	
U6	5′-CAGCACATATACTAAAATTGGAACG-3′	76 bp
	5′-ACGAATTTGCGTGTCATCC-3′	

Supplementary Table S2: List of antibodies used for western blotting

Antibody	Host	Supplier	Product No.	Working Dilution
anti-PPM1F	Mouse	Santa Cruz Biotechnology	J1311	1:1000
anti-MLC2	Rabbit	Cell Signaling Technology	#3672	1:1000
anti-pMLC2 (Thr18/Ser19)	Rabbit	Cell Signaling Technology	#3674	1:1000
anti-PDGFRA	Rabbit	Epitomics	#3938-1	1:1000
anti-SP1	Rabbit	Epitomics	#5407-1	1:1000
anti-YWHAZ	Rabbit	Epitomics	#5175-1	1:10000
anti-RAP1	Rabbit	Epitomics	#2291-1	1:500
anti-CD47	Rabbit	Epitomics	#3847-1	1:1000
HRP-coupled anti-mouse IgG	Goat	Santa Cruz Biotechnology	sc-2055	1:10,000
HRP-coupled anti-rabbit IgG	Goat	Santa Cruz Biotechnology	sc-2005	1:10,000
